# Temporal Association between Abdominal Weight Status and Healthy Aging: Findings from the 2011–2018 National Health and Aging Trends Study

**DOI:** 10.3390/ijerph17165656

**Published:** 2020-08-05

**Authors:** Furong Xu, Jacob E. Earp, Geoffrey W. Greene, Steven A. Cohen, Ingrid E. Lofgren, Matthew J. Delmonico, Mary L. Greaney

**Affiliations:** 1 Department of Kinesiology, University of Rhode Island, Independence Square II, Kingston, RI 02881, USA; jacob_earp@uri.edu (J.E.E.); delmonico@uri.edu (M.J.D.); 2 Department of Nutrition and Food Sciences, University of Rhode Island, Fogarty Hall, Kingston, RI 02881, USA; ggreene@uri.edu (G.W.G.); ingrid_lofgren@uri.edu (I.E.L.); 3 Department of Health Studies, University of Rhode Island, Independence Square II, Kingston, RI 02881, USA; steven_cohen@uri.edu (S.A.C.); mgreaney@uri.edu (M.L.G.)

**Keywords:** healthy aging, waist circumference, abdominal obesity

## Abstract

Background/objectives: The longitudinal effect of abdominal weight status (AWS) defined by waist circumference (WC) on healthy aging has not yet been comprehensively examined. Therefore, the purpose of the present study was to examine the temporal association between WC-defined AWS and a comprehensive assessment for healthy aging. Subjects/methods: This study utilized data from 5211 respondents aged 65+ who participated in the National Health and Aging Trends Study from 2011 to 2018. Mixed effects regression models were used to examine the association between baseline AWS and the annual change rate in healthy aging score (HAS) via interaction terms (AWS*round) adjusting for confounding effects. Further multiple mixed models examined the relationship of AWS and HAS over an 8-year period. Results: There were no annual change rate differences in HAS by baseline AWS, regardless of sex. However, males with abdominal obesity were more likely to have a lower HAS than males with normal AWS (β = −0.20, 95% CI: −0.30, −0.10, *p* < 0.001) but no difference in HAS was observed between males with overweight and normal AWS. A similar pattern was observed among females. Conclusions: Study results indicate that AWS was associated with HAS but it did not modify annual HAS change rate over time.

## 1. Introduction

The United States (US) population is steadily aging. The percentage of adults aged 65+ increased 34.2% over the past decade [1] and thus it is important to understand predictors of healthy aging in older adults. Healthy aging is a complex and multifactorial concept that incorporates factors associated with the aging process, including physical, mental, and social wellbeing [2,3]. The World Health Organization (WHO) describes healthy aging as the absence of major chronic diseases (e.g., diabetes, cancer, cardiovascular disease), the presence of good physical/cognitive function, and wellbeing [2]. Recent research indicates that additional health indicators are important for healthy aging, such as health-related limitations in social life, function limiting pain, mental health, and perceived health status [3,4,5,6]. Despite efforts to identify predictors of healthy aging, existing longitudinal studies have utilized only a limited range of health indicators to measure healthy aging and have not incorporated other factors such as major chronic diseases [3], wellbeing [5,6], function limiting pain [3,6], and perceived health [4,6]. To better understand factors associated with healthy aging, there is a critical need for longitudinal research that comprehensively measures healthy aging and considers key health indicators identified from the existing literature.

The relationship between weight status defined by body mass index (BMI) and aging has been studied extensively [7,8]. Even though existing longitudinal studies have primarily focused on a limited number of aging-related health indicators such as physical function, disability, dementia, and comorbidities, these studies provide critical evidence of the relationships between weight status categories based on BMI and the health of older adults [7,8]. However, in recent years, waist circumference (WC) has been recognized as a better measure than BMI in older adults for longitudinal aging studies [9]. The reason for this is that older adults are likely to experience age-related decreases in muscle and bone mass and BMI weight classifications would view these as healthy changes while abdominal weight status (AWS) defined by WC would not and it was less influenced by aging than BMI [10,11]. In addition, BMI does not distinguish abdominal obesity, which is the most metabolically deleterious, whereas WC directly reflects abdominal fat mass [9,10]. Accordingly, the purpose of the present study was to examine associations between AWS defined by WC and a comprehensive assessment of healthy aging over an eight-year period in older adults aged 65+ in the US using nationally representative longitudinal data. All analyses were stratified by sex due to the sex difference identified in the existing literature [12].

## 2. Method

The present study (see Supplementary Table S1 for study design summary) used data drawn from the 2011–2018 National Health and Aging Trends Study (NHATS), which was a longitudinal study of nationally representative Medicare beneficiaries aged 65+ years in the USA NHATS conducted surveys and measurements annually with response rates ranging from 70.9% to 94.8% [13,14]. A total of 8245 individuals participated in 2011 NHATS and 8077 of these had positive analytic weight [15]. Respondents were excluded from the current study for the following reasons: (1) missing baseline WC and BMI data (*n* = 1477) in 2011; (2) BMI < 18.5 kg/m^2^ in any round of data collection (*n* = 436) due to the possible underweight related physical and psychological pathology [16]; and (3) missing baseline health indicators (see method for detailed information) related to healthy aging (*n* = 953). Using these criteria, 5211 respondents were included in the present study, which was approved by the Institutional Review Board of the University of Rhode Island (IRB# 1551268-1).

### 2.1. Abdominal Weight Status

The present study utilized WC to determine AWS. At baseline and each of the following seven rounds, WC was measured by trained staff using a flexible tape measure around each respondent’s waist at the level of their umbilicus following standardized protocol [17]. WC was used to create the following AWS categories: normal (males WC < 37 inches, females WC < 31.5 inches), overweight (males 37 inches ≤ WC < 40 inches, females 31.5 inches ≤ WC < 35 inches), and obese (males WC ≥ 40 inches, females WC ≥ 35 inches) [9,18].

### 2.2. Healthy Aging Score (HAS)

Healthy aging was assessed using 10 health indicators based on the WHO and Assmann and colleagues’ definition of healthy aging [2,5]. These indicators (see Table 1) encompassed physical and cognitive function, wellbeing, major chronic disease, depression, anxiety, instrumental activities of daily living (IADL), health-related limitations in social life, function limiting pain, and perceived health [2,5]. Each health indicator was scored “1” if the established or set criterion was met and “0” if it was not. Scores for each indicator were then summed to create a HAS (range 0–10). The HAS was then dichotomized based on score distribution as done in prior healthy aging research [6]: (1) good (above median, scores 7–10) and (2) poor (below median, scores 0–6). Each of the 10 health indicators and the scoring criteria are discussed below.

Physical function was measured by the short physical performance battery (SPPB), which is comprised of three subtests: balance stand, walking speed, and repeated chair stand [14,17]. SPPB scores were calculated using the NHATS’ SAS programming statements and range 0–12 [19]. Respondents were classified as meeting the physical function criterion if their SPPB total score was 10–12 [20,21].

The presence of cognitive impairment was identified by utilizing three measures: (1) previous diagnosis of dementia or Alzheimer’s disease (AD), (2) the AD8 dementia screening interview, and (3) cognitive tests of memory, orientation, and executive function [22]. The NHATS’ SAS program statement was used to calculate cognition impairment scores and dementia classifications [23,24]. Respondents were classified as meeting the criterion of no cognitive impairment/dementia if there had been no reported diagnosis of dementia or AD, did not meet the AD8 criteria, and were not identified as having impairment in any of the cognitive tests [22].

Wellbeing was assessed by 11 items adapted from the National Survey of Midlife Development in the USA, which measured three aspects of wellbeing: positive and negative affect (4 items), sense of control (4 items), and psychological wellbeing (3 items) [15]. Wellbeing scores for the current study were based on criteria used in previous research [25]. Since no cut points are available for wellbeing, respondents’ scores were divided into tertiles (1) poor (scored 1–33), (2) fair (scored 34–37), and (3) good (scored 38–41). Wellbeing scores categorized as good were considered to be indicative of good wellbeing and meeting criterion.

The presence of major chronic disease was assessed by three items that asked respondents if they had been diagnosed with heart disease, diabetes, or cancer [5,14]. Respondents who answered “no” to all of these questions were classified as having no major chronic disease and as meeting the criterion [5,14].

Depression was assessed by the patient health questionnaire-2, which included two items asking respondents how frequently in the previous month they had “little interest or pleasure in doing things” or “felt down, depressed, or hopeless” [14]. Depression scores ranged from 0 to 6 and scores less than 3 were classified as meeting the criterion of no depression [14,26].

Anxiety was assessed by generalized anxiety disorder-2, which included two items assessing respondents’ frequency in the last 30 days regarding “felt nervous, anxious, or on edge” and “been unable to stop or control worrying”. Anxiety scores ranged from 0 to 6 and respondents were classified as meeting the criterion of no anxiety if they scored less than 3 [14,27].

The IADL limitations was measured via a validated instrument that asked respondents to identify their difficulty (none, a little, some, a lot) in completing five household activities (medication tracking, doing laundry, groceries shopping, making hot meals, handling bills, and banking) or the reasons why these activities were done by or with someone else [28]. Respondents were classified as having no IADL limitations if they had no difficulty carrying out these five activities or if these activities were done by or with someone else due to reasons that were not due to respondent’s health or functioning [5,28].

Health-related limitations in one’s social life were assessed by 10 questions about participation in social events in the last month and whether participation was limited due to health reasons [14,28]. Respondents were asked if their health or functioning in the past 30 days ever kept them from doing the following activities that were somewhat or very important to them: (1) visiting with friends and family; (2) attending religious services; (3) participating in clubs, classes, or other organized activities; or 4) going out to dinner, a movie, or a musical/theatrical performance. Respondents were classified as having no health-related limitation in their social life if they reported to having no restrictions with regard to any activities that was claimed as “somewhat important’ or “very important” to them [28].

Function limiting pain was assessed by three items. Respondents were classified as having no function limiting pain if they were not bothered by pain and pain had not limited their activities, or if they reported that they “rarely or never” took pain medication in the last month [5,17].

Overall perceived health status was assessed by one item that asked participants to rate their health status (excellent, very good, good, fair, or poor) [14]. Respondents were classified as meeting criterion if they assessed their healthy status as being “excellent”, “very good”, or “good” [5].

### 2.3. Demographic Characteristics

The demographic characteristics were examined including age (65–74, 75–84, 85+), sex (male, female), race/ethnicity (White, Black, Hispanic, Others), education (high school or less, college or above), annual income (<$27,600, $27,600–41,999, $42,000–63,999, $64,000–107,999, ≥$108,000), and homebound status (homebound, semi-homebound, not homebound) [14]. Homebound status was assessed by three variables: frequency, help needed, and difficulty going outside in the last 30 days [14,29].

### 2.4. Data Analysis

Analytic weights were applied to all analyses, as suggested by NHATS, to reduce possible sample bias [30,31]. For baseline sample characteristics, continuous and categorical variables were tabulated by weighted mean ± standard errors and frequencies and weighted proportions (%), respectively. Differences in respondent characteristic by HAS categories (good versus poor) were examined by performing linear regression or logistic regression models. Time trend plots were performed to check the pattern of change in HAS longitudinally by baseline AWS, whereas *p*-values for trends and interaction terms (AWS*round) were calculated using a univariate mixed effect regression model for HAS accounting for correlation with repeated measures and utilization of weighted data with the analytic weight, which adjusts for loss to follow-up. Round was treated as a continuous variable.

Additionally, adjusted βs (95% confidence intervals (CIs)) and *p*-values for HAS trends by baseline AWS were estimated from three mixed effects regression models to look at annual HAS change rates for three AWS categories, respectively. Adjusted odds ratios (95% CIs) and *p*-values were estimated using three generalized estimating equation models to look at the proportion of annual HAS category change rates for three AWS categories, respectively. Every model accounted for clustering and round was treated as a continuous variable, adjusted for age, race/ethnicity, education, annual income, and homebound status. Then, the interaction terms stratified variables*round was added into the model to examine the interaction between AWS and round, as well as to investigate whether the annual changes in HAS and HAS categories differed by baseline AWS. The mixed regression model represents an outcome variable (HAS) as a function of an intercept (β_0_), the predictor variable (AWS), and a random error term (intercepts and slopes). The model was specified as follows: HAS_ij_ = β_0j_ + β_1j_ x round + β_2_j AWS + β_3j_ AWS x round + β_ij_ x covariates + E_ij_, where HAS_ij_ represents the HAS value for person (i) at time (j); β_0j_ (= γ_00_ + u_0j_) represents the person-specific intercept or baseline HAS value; γ_00_ represents the fixed average intercept across all individuals; u_0j_ is random effect term; β_1_j (= γ_10_ + u_1j_) represents the person-specific slope of change in HAS over round; β_2j_ represents the person-specific slope of change in HAS with the change of AWS; β_3j_ represents the relationship between AWS and the rate of change in HAS over round, which represents the cross-level interaction between the level 1 (within-subjects) variable, round, and level 2 (between subjects) variable, AWS; E_ij_ represents the residual error or deviation of the observed HAS values for each person (i) at time (j).

Furthermore, the temporal associations of AWS and healthy aging were examined. For HAS outcomes, β (95% CIs) and *p*-values were estimated from mixed models; for HAS categorical outcomes, odds ratios (95% CIs) and *p*-values were estimated from generalized estimating equation models. All models accounted for the correlation with repeated measures. All multiple models were adjusted for age, race/ethnicity, education, annual income, homebound status, and round. All statistical analyses were stratified by sex except descriptive analysis of demographic characteristics and conducted using SAS 9.4 (SAS Institute Inc., Cary, NC, USA) and *p* < 0.05 was considered to be statistically significant.

## 3. Results

The study sample was 54.8% females wherein 9.5% were 85+ years of age. The sample was primarily whites with only 16% being classified as an ethnic minority. In addition, 18.3% of the sample had a high school degree or less, 37.3% had an annual income of less than $27,600, and 2.5% were homebound. A majority of respondents (69%) were categorized as having abdominal obesity while 11.4% had normal AWS. There was a difference in HAS score with AWS categories for normal and overweight respondents more likely to be categorized as having good HAS, while respondents with abdominal obesity was more likely to be categorized as having poor HAS (see Table 2).

**Figure 1:**
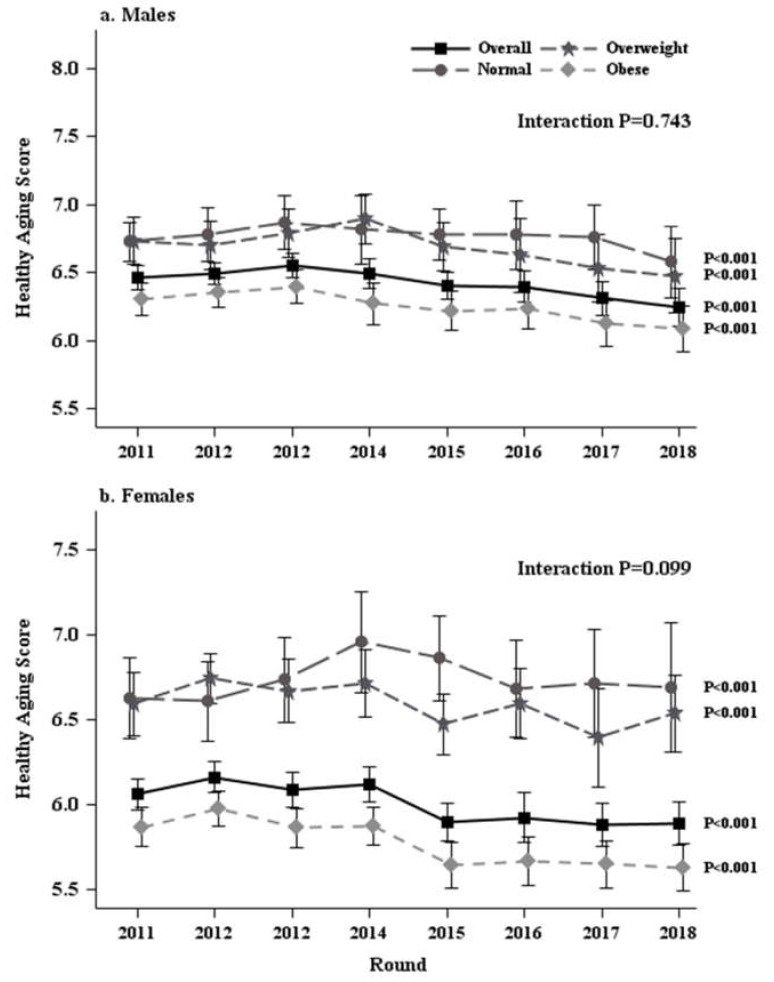
Healthy aging score trends by baseline abdominal weight status, National Health and Aging Trends Study 2011–2018.

After examining differences in demographics at baseline, the next step of the analyses was to examine HAS trends by baseline AWS in males and females, respectively, over eight years. Results indicated that the decline in HAS was significant (all *p* < 0.001) (see Figure 1 and Supplementary Table S2). There was also a significant increase in the proportion of respondents who classified as having poor HAS (*p* < 0.001). At baseline, 44.3% of males and 54% of females were classified as having poor HAS, whereas 57.3% of males and 65.3% of females were classified as having poor HAS after eight years (see Supplementary Table S2). Furthermore, as shown in Supplementary Table S2, respondents with abdominal obesity started with lower HAS (6.31 for obese vs. 6.73 for both normal and overweight in males; 5.87 for obese vs. 6.63 and 6.59 for normal and overweight in females) and remained lower than those with normal and overweight AWS over 8 years regardless of sex. Similarly, a higher proportion of respondents with abdominal obesity were classified as having poor HAS than those with normal and overweight AWS at baseline (47.2% for obese vs. 37.5% and 40.4% for normal and overweight in males; 59.2% for obese vs. 37.3 % and 40.4% for normal and overweight in females) and remained higher in the proportion of poor HAS after eight years (62.3% for obese vs. 42.1% and 52.1% for normal and overweight in males; 71.1% for obese vs. 38.9 % and 54.4% for normal and overweight in females) (see Supplementary Table S2).

The analyses then examined annual change rate in the HAS and HAS categories stratified by baseline AWS over eight years (Table 3). For males whose AWS was classified as normal, for every 1-year increase in age (each round), the HAS decreased by 0.09 (β = −0.09, 95% CI: −0.12, −0.06), and the odds of the proportion of respondents being classified as having poor HAS increased by 16% (OR = 1.16, 95% CI; 1.11–1.22). Similar patterns were observed for males whose AWS was classified as overweight (HAS: β = −0.09, 95% CI: −0.11, −0.07; poor HAS: OR = 1.14, 95% CI: 1.09, 1.19) and obese (HAS: β = −0.10, 95% CI: −0.11, −0.08; poor HAS: OR = 1.15, 95% CI: 1.12, 1.18), as well as in females regardless of AWS classifications. However, there was no significant annual change rate differences by AWS in HAS and HAS categories regardless of sex.

The analyses then examined the temporal association between AWS and HAS, utilizing data from all eight rounds of data collection. As shown in Table 4, the HAS for male respondents with abdominal obesity was lower than male respondents with normal AWS (β = −0.20, 95% CI: −0.30, −0.10). Results were similar in females. Respondents with abdominal obesity had lower HAS than those with normal AWS (β = −0.15, 95% CI: −0.24, −0.05). There was no difference in HAS between respondents with abdominal overweight and normal AWS.

While examining the temporal association between AWS and the HAS categories that utilized AWS and HAS data from all rounds (see Table 4), male respondents who were overweight (OR = 1.26, 95% CI: 1.08, 1.48) or obese (OR = 1.52, 95% CI: 1.29, 1.79) were more likely to be classified as having poor HAS than respondents with normal AWS. For females, respondents who were overweight (OR = 1.16, 95% CI: 1.01, 1.35) or obese (OR = 1.42, 95% CI: 1.21, 1.66) were also more likely to be classified as having poor HAS than those with normal AWS.

## 4. Discussion

This is the first study, to our knowledge, that has examined the temporal associations between AWS defined by WC and a comprehensive assessment of healthy aging in a nationally representative sample of older adults in the US. Study findings indicate that HAS in all AWS categories decreased annually but the rate of annual HAS decline in respondents with overweight or obese AWS was not significantly different than those with normal AWS. However, respondents with abdominal obesity always had lower HAS, and respondents whose AWS was classified as overweight and obese were more likely to have poor HAS than those with normal AWS.

While a number of previous studies investigated health indicators among older adults—such as physical function and dementia [7,8,9,32,33,34,35]—the present study advances previous research by creating a more comprehensive measure of healthy aging that included measures of physical/cognitive function, wellbeing, major chronic diseases, mental health, function limitation in household activities, and health-related limitations in social life, function limiting pain, and perceived health status [2,5]. When HAS were evaluated longitudinally, they decreased steadily regardless of AWS and sex. Although annual decrease in HAS (ranging from 0.08 to 0.10) is relatively small, the accumulation of this change overtime could have a significant impact on the trajectory of healthy aging. This finding aligns somewhat with prior research that examined certain aspects of healthy aging, such as limitations in social life, cognitive impairment, IADL, and pain [32,33,35,36]. However, it is difficult to make comparisons between the current study and previous studies due to different analytical approaches, as the current study examined annual change rate of the comprehensive assessment of healthy aging whereas previous studies did not. Nevertheless, the present study adds to the literature with important information on a comprehensive assessment of HAS changes over an eight-year period by baseline AWS in a nationally representative sample of adults aged 65+ in the US. Moreover, the use of WC is a unique strength of the present study. WC is a surrogate marker of central adiposity while BMI is simply a weighted ratio of height and body mass [9,10]. Previous studies have reported that WC is a marker of visceral fat associated with cardiometabolic risk [37], and that WC is also more closely related to morbidity and mortality in older adults than BMI [9]. This distinction is particularly important as older adults are at risk for sarcopenia-related muscle loss, which results in a loss of functional mass due to aging [11]. In addition, older adults are at risk for bone loss, which contributes to a loss of functional mass related to aging [10]. Both muscle and bone loss could result in healthier weight classification based on BMI despite increased relative adiposity and mortality [10,11]. Nonetheless, due to these differences between WC and BMI, caution needs to be applied when comparing the present study’s findings to findings of studies that used BMI as opposed to WC.

The present study is also unique in its use of longitudinal AWS data while examining its associations with healthy aging overtime. AWS is important although AWS does not modify HAS annual change rate overtime. Respondents with abdominal obesity were more likely to have a lower HAS and to be classified as having poor HAS than those with normal AWS, regardless of sex. This finding is in agreement with previous studies [7,8,38] and the current study adds to the literature by examining the longitudinal association between AWS defined by WC and a comprehensive assessment of healthy aging. The high proportion of respondents (69%) classified as obese by AWS and the deleterious effect of this classification on HAS, indicate the need for strategies to prevent abdominal fat accumulation in older adults. It is worth noting that although there was no significant difference in HAS between respondents with overweight and normal AWS, respondents with overweight AWS were more likely to be classified as having poor HAS than those with normal AWS. This finding is not consistent with studies that used BMI to determine weight status that found being overweight is associated with lower mortality risk in older adults [39]. Nevertheless, the possible explanation for the difference observed in poor HAS classification between respondents with overweight and normal AWS is that being abdominally overweight after age 65 might still pose a risk to healthy aging in older adults. Difference may also be due to the precision of measurement although in this study, WC measurement was conducted by trained assessors using the standardized protocol [17]. Further research is warranted to examine possible healthy aging related factors in a large cohort of older adults to better understand the differential effect of AWS and BMI categorization on the trajectory of healthy aging.

The present study has several strengths. First, to our knowledge, it is the first study to utilize a comprehensive definition of healthy aging to examine healthy aging trends and its association with AWS in older adults using representative data in the US. The longitudinal data collected over eight years allowed us to examine the holistic aspect of healthy aging overtime. In addition, the present study used WC to define AWS, which is a more appropriate measure in older adults [9]. Moreover, the present study adjusted for important confounders that have been identified as risk factors, including homebound status that has not been adjusted in prior research [14,29]. However, there could be other residual confounding factors not included that might bias our results. Some of the criteria used to create the HAS were based on self-reported measures (e.g., anxiety, depression, IADL), although these measures were validated [25,26,27,28]. Another study limitation is that WC is not as accurate as dual energy X-ray absorptiometry when measuring abdominal visceral fat [40]. However, WC has consistently been associated with abdominal fat measured via dual energy X-ray absorptiometry in older adults [41] and is an efficient and cost-effective measurement for general clinical assessment as well as for use with large cohort studies [9]. Furthermore, 84% of the sample were white, which presents a limitation for the generalization of our findings to a broader population. It is also possible that our sample might be skewed to those who were healthier and lived longer.

## 5. Conclusions

Findings from the present study indicated that AWS was associated with healthy aging but did not modify the annual rate of change for HAS overtime. However, these findings still highlighted the importance of AWS because respondents with abdominal obesity had lower HAS at all points over an eight-year period compared to respondents with normal AWS. Results also indicated that both respondents with overweight and obese AWS were more likely to be categorized as having poor HAS than respondents with normal AWS. These findings indicate that abdominal obesity decreases the likelihood for successful aging and the effect of having overweight AWS on healthy aging is inconsistent. Study results suggest that, moving forward, studies examining weight in older adults should use WC defined weight status rather than BMI.

## Figures and Tables

**Table 1:** Health indicators and its criteria for the definition of healthy aging.

Health Indicator	Met Criteria (Score of 1)	Not Met Criteria (Score of 0)
Physical function	SPPB = 10–12	SPPB < 10
Cognitive impairment	No cognitive impairment or dementia	Cognitive impairment or dementia
Wellbeing	Scored 38–41 (3rd tertile)	<38 (1st and 2nd tertiles)
Major chronic disease	No heart disease, diabetes, or cancer	Had 1 or more major chronic diseases (heart disease, diabetes, cancer)
Depression	PHQ-2 scored < 3	PHQ-2 scored ≥ 3
Anxiety	GAD-2 scored < 3	GAD-2 scored ≥ 3
IADL limitations	No IADL limitation	Had 1 or more IADL limitations
HRLS	No HRLS	Had HRLS
Function limiting pain	No function limiting pain	Had function limiting pain
Perceived overall health	Excellent, very good, or good	Fair or poor

Note: SPPB = short physical performance battery; PHQ = patient health questionnaire; GAD = generalized anxiety disorder; IADL = instrumental activity of daily life; HRLS = health-related limitation in social life.

**Table 2:** Baseline characteristics of respondents stratified by HAS classification, NHATS 2011.

Variables	Total	Good HAS ^$^	Poor HAS ^$^	*p*-Value
	*n* = 5211	*n* = 2369 (50.4%)	*n* = 2842 (49.6%)	
Females, *n* (weighted%)	2916 (54.8)	1196 (50.1)	1720 (59.6)	<0.001 *
Age classification, *n* (weighted %)				
65–74 yrs	2299 (56.9)	1233 (64.0)	1066 (49.8)	<0.001 *
75–84 yrs	2100 (33.5)	894 (30.2)	1206 (37.0)	<0.001 *
85+ yrs	812 (9.5)	242 (5.8)	570 (13.2)	<0.001 *
Race/ethnicity, *n* (weighted %)				
White	3770 (84.0)	1836 (87.2)	1934 (80.7)	<0.001 *
Black	1048 (7.4)	386 (5.8)	662 (9.0)	<0.001 *
Hispanic	269 (6.0)	87 (4.3)	182 (7.7)	<0.001 *
Others	115 (2.6)	55 (2.6)	60 (2.6)	0.962
Education, *n* (weighted %)				
High school or less	1177 (18.3)	350 (12.6)	827 (24.1)	<0.001 *
College or above	4032 (81.7)	2017 (87.4)	2015 (75.9)	<0.001 *
Annual income, *n* (weighted %)				
<$27,600	2293 (37.3)	723 (25.7)	1570 (49.1)	<0.001 *
$27,600–$41,999	975 (18.7)	451 (18.2)	524 (19.1)	0.424
$42,000–$63,999	838 (17.5)	475 (20.2)	363 (14.7)	<0.001 *
$64,000–$107,999	716 (17.0)	455 (22.6)	261 (11.4)	<0.001 *
≥ $108,000	389 (9.5)	265 (13.3)	124 (5.7)	<0.001 *
Homebound status, *n* (weighted %)				
Homebound	174 (2.5)	9 (0.2)	165 (4.8)	<0.001 *
Semi-homebound	238 (3.7)	14 (0.4)	224 (7.0)	<0.001 *
Not homebound	4799 (93.8)	2346 (99.4)	2453 (88.2)	<0.001 *
WC, inches	39.6 ± 0.2	38.7 ± 0.2	40.5 ± 0.2	<0.001 *
Abdominal weight status, *n* (weighted %)				
Normal ^#^	600 (11.4)	345 (14.2)	255 (8.6)	<0.001 *
Overweight ^#^	1003 (19.6)	552 (23.1)	451 (15.9)	<0.001 *
Obese ^#^	3608 (69.0)	1472 (62.7)	2136 (75.4)	<0.001 *
HAS (0–10)	6.2 ± 0.0	7.7 ± 0.0	4.8 ± 0.0	<0.001 *
Health indicators met criteria, *n* (weighted %)				
Met physical function criterion	2444 (55.1)	1824 (82.9)	620 (27.0)	<0.001 *
Met no cognitive impairment/no dementia criterion	4324 (86.8)	2220 (95.1)	2104 (78.3)	<0.001 *
Met good well-being criterion	1591 (31.5)	1260 (51.8)	331 (10.8)	<0.001 *
Met no major chronic diseases criterion	2459 (48.7)	1587 (66.9)	872 (30.2)	<0.001 *
Met no depression criterion	4555 (88.5)	2324 (98.0)	2231 (78.8)	<0.001 *
Met no anxiety criterion	4651 (89.8)	2348 (99.0)	2303 (80.4)	<0.001 *
Met no IADL limitation criterion	329 (5.4)	114 (4.4)	215 (6.5)	<0.001 *
Met no HRLS criterion	4353 (85.5)	2329 (98.4)	2024 (72.5)	<0.001 *
Met perceived overall health criterion	3974 (79.7)	2315 (98.3)	1659 (60.9)	<0.001 *
Met no function-limiting pain criterion	2745 (53.2)	1849 (76.3)	896 (29.8)	<0.001 *

Note: continuous and categorical variables were tabulated by weighted mean ± standard errors and frequencies and weighted proportions (%), *p*-values for continuous variables obtained by performing linear regression model, whereas logistic regression model for categorical variables. NHATS = National Health and Aging Trends Study; IADL= instrumental activities of daily living; HRLS = health-related limitation in social life; HAS = healthy aging score. ^$^ Good HAS, defined as above the median; poor HAS defined as below the median. WC = waist circumference: # normal (WC < 37 inches/31.5 inches), overweight (37 inches/31.5 inches ≤ WC < 40 inches 35 inches), and obese (WC ≥ 40 inches/35 inches) in males and females, respectively, * *p* < 0.05.

**Table 3:** Annual changes in HAS and categories stratified by baseline AWS, NHATS 2011–2018.

Variables	Total HAS	Good HAS ^$^	Poor HAS ^$^	
	Adjusted β (95% CI), *p* for Trend ^&^	Adjusted OR (95% CI), *p* for Trend ^@^		
Males stratified by AWS				
Normal	−0.09 (−0.12, −0.06), <0.001 *	0.86 (0.82, 0.90), <0.001 *	1.16 (1.11, 1.22), <0.001 *	
Overweight	−0.09 (−0.11, −0.07), <0.001 *	0.88 (0.84, 0.92), <0.001 *	1.14 (1.09, 1.19), <0.001 *	
Obese	−0.10 (−0.11, −0.08), <0.001 *	0.87 (0.85, 0.89), <0.001 *	1.15 (1.12, 1.18), <0.001 *	
Interaction terms AWS *round ^#^	0.781	0.772	0.772	
Normal	REF	REF	REF	
Overweight	0.000 (−0.036, 0.036), 0.997	1.02 (0.96, 1.09), 0.48	0.98 (0.92, 1.04), 0.48	
Obese	−0.008 (−0.040, 0.024), 0.627	1.01 (0.96, 1.07), 0.617	0.99 (0.93, 1.04), 0.617	
Females stratified by AWS				
Normal	−0.08 (−0.11, −0.05), <0.001 *	0.91 (0.86, 0.96), <0.001 *	1.10 (1.04, 1.16), <0.001 *	
Overweight	−0.08 (−0.10, −0.06), <0.001 *	0.89 (0.85, 0.93), <0.001 *	1.12 (1.07, 1.18), <0.001 *	
Obese	−0.10 (−0.12, −0.09), <0.001 *	0.89 (0.87, 0.91), <0.001 *	1.13 (1.10, 1.15), <0.001 *	
Interaction terms AWS *round^#^	0.102	0.588	0.588	
Normal	REF	REF	REF	
Overweight	−0.006 (−0.045, 0.033), 0.751	0.98 (0.91, 1.04), 0.467	1.03 (0.96, 1.10), 0.467	
Obese	−0.027 (−0.061, 0.006), 0.113	0.97 (0.92, 1.03), 0.295	1.03 (0.97, 1.09), 0.295	

Note: ^&^ adjusted β (95% confidence intervals (CIs)) CI) and *p* for trend were estimated from mixed effects regression models. ^@^ Adjusted odds ratio (OR) and *p* for trend were estimate using generalized estimating equation models; all the models accounted for clustering and round was treated as a continuous variable (1–8) adjusted by age, race/ethnicity, education, annual income, and homebound status. ^#^ The interaction terms stratified variables*round was added into the model to examine the effect of the interaction between stratified variables and round to investigate whether the changes over years in prevalence or mean differed between the stratified variables. NHATS = National Health and Aging Trends Study; AWS= abdominal weight status; HAS = healthy aging score. ^$^ Good HAS defined as above the median whereas poor HAS defined as below the median; * *p* < 0.05.

**Table 4:** Temporal associations between AWS and HAS, NHATS 2011–2018.

Variables	Total HAS	Poor HAS vs. Good HAS ^$^
	β (95% CI), *p*-Value ^#^	OR (95% CI), *p*-Value ^&^
Males stratified by AWS		
Normal	REF	REF
Overweight	−0.05 (−0.15, 0.04), 0.265	1.26 (1.08, 1.48), 0.004 *
Obese	−0.20 (−0.30, −0.10), <0.001 *	1.52 (1.29, 1.79), <0.001 *
Females stratified by AWS		
Normal	REF	REF
Overweight	−0.05 (−0.14, 0.05), 0.328	1.16 (1.01, 1.35), 0.049 *
Obese	−0.15 (−0.24, −0.05), 0.002 *	1.42 (1.21, 1.66), <0.001 *

Note: ^#^ β (95% confidence intervals (CIs)) and *p*-values were estimated from mixed models; ^&^ odds ratios (ORs) (95% CIs) and *p*-values were estimated from generalized estimating equation models. All models were accounted for the correlation with repeated measures, and all analyses adjusted for age, race, education, annual income, homebound status, and round. NHATS = National Health and Aging Trends Study; AWS = abdominal weight status; HAS = healthy aging score. ^$^ Good HAS defined as above the median whereas poor HAS defined as below the median, * *p* < 0.05.

## Supplementary Materials

The following are available online at https://www.mdpi.com/1660-4601/17/16/5656/s1, Table S1: Study design summary, Table S2: Healthy aging score trend by baseline AWS, NHATS 2011–2018.
