# Supplementary material for: Temporal Association between Abdominal Weight Status and Healthy Aging: Findings from the 2011–2018 National Health and Aging Trends Study
Source: Int J Environ Res Public Health. 2020 Aug 5;17(16):5656. doi: 10.3390/ijerph17165656 (PMC7459859; doi:10.3390/ijerph17165656)
Supplement: Supplementary file 1 [file ijerph-17-05656-s001.pdf]

Supplementary Table S1. Study design summary.

Objective	Exposure variables	Outcomes	Measurement method	Time period	Statistical analysis
To examine the temporal association between AWS defined by WC and healthy aging	AWS<br><br>Age, race/ethnicity, education, annual income, homebound status	HAS<br><br>HAS status (good vs. poor)	Secondary data - NHATS<br><br>HAS was created utilizing ten health indicators<br><br>HAS status was based on respondents' HAS score distribution	8 years	The associations between baseline AWS and the annual change rate in HAS were estimated from multiple mixed effect regression models via interaction terms<br><br>The relationships between AWS and HAS over 8 years were examined using mixed models

Note: AWS = Abdominal weight status; WC= Waist Circumference; HAS = Healthy aging score; NHATS= National Health and Aging Trends Study.

Supplementary Table S2. Healthy aging score trends by baseline AWS, NHATS 2011-2018.

	N	HAS total@	Good HAS&, #	Poor HAS&, #
**Males**				
Total		**Mean (95% CI)**	**Prevalence (95% CI)**	**Prevalence (95% CI)**
2011	2295	6.46 (6.37 - 6.55)	55.7 (55.1 - 56.2)	44.3 (43.8 - 44.9)
2012	1660	6.49 (6.41 - 6.57)	50.0 (49.5 - 50.4)	50.0 (49.5 - 50.6)
2013	1294	6.55 (6.47 - 6.64)	46.9 (46.4 - 47.4)	53.1 (52.6 - 53.6)
2014	1044	6.49 (6.38 - 6.60)	45.4 (44.8 - 46.0)	54.6 (54.1 - 55.1)
2015	1027	6.40 (6.30 - 6.50)	44.8 (44.6 - 45.0)	55.2 (55.0 - 55.4)
2016	892	6.40 (6.28 - 6.51)	44.5 (44.3 - 44.8)	55.5 (55.2 - 55.7)
2017	815	6.31 (6.18 - 6.44)	43.3 (43.0 - 43.5)	56.7 (56.5 - 57.0)
2018	732	6.25 (6.11 - 6.39)	42.7 (42.4 - 43.0)	57.3 (57.0 - 57.6)
P for trend		<0.001*	<0.001*	<0.001*
Normal				
2011	352	6.73 (6.58 - 6.87)	62.5 (61.6 - 63.4)	37.5 (36.6 - 38.4)
2012	255	6.78 (6.59 - 6.98)	57.1 (55.9 - 58.3)	42.9 (41.5 - 44.3)
2013	198	6.87 (6.68 - 7.06)	53.9 (52.7 - 55.1)	46.1 (44.8 - 47.4)
2014	153	6.82 (6.56 - 7.07)	50.8 (49.2 - 52.5)	49.2 (47.5 - 50.8)
2015	148	6.78 (6.59 - 6.97)	51.4 (50.8 - 52.0)	48.6 (48.1 - 49.2)
2016	126	6.78 (6.52 - 7.03)	51.3 (50.7 - 52.0)	48.7 (48.1 - 49.2)
2017	121	6.76 (6.52 - 7.00)	53.7 (53.2 - 54.2)	46.3 (45.7 - 46.9)
2018	115	6.58 (6.31 - 6.84)	57.9 (57.4 - 58.5)	42.1 (41.5 - 42.6)
P for trend		<0.001*	<0.001*	<0.001*
Overweight				
2011	531	6.73 (6.56 - 6.91)	59.6 (58.5 - 60.7)	40.4 (39.3 - 41.5)
2012	388	6.70 (6.52 - 6.88)	53.7 (52.8 - 54.6)	46.3 (45.1 - 47.4)
2013	299	6.79 (6.61 - 6.97)	53.9 (52.8 - 55.0)	46.1 (44.9 - 47.2)
2014	245	6.90 (6.71 - 7.08)	53.9 (52.8 - 55.0)	46.1 (45.0 - 47.1)
2015	233	6.69 (6.51 - 6.87)	51.4 (51.0 - 51.8)	48.6 (48.2 - 49.0)
2016	205	6.63 (6.36 - 6.90)	50.8 (50.2 - 51.3)	49.2 (48.7 - 49.7)
2017	189	6.53 (6.28 - 6.78)	47.3 (46.7 - 47.8)	52.7 (52.2 - 53.3)
2018	167	6.48 (6.20 - 6.75)	47.9 (47.3 - 48.5)	52.1 (51.6 - 52.6)
P for trend		<0.001*	<0.001*	<0.001*
Obese				
2011	1412	6.31 (6.19 - 6.43)	52.8 (52.1 - 53.4)	47.2 (46.6 - 47.9)
2012	1017	6.35 (6.25 - 6.46)	47.0 (46.3 - 47.7)	53.0 (52.3 - 53.7)
2013	797	6.40 (6.27 - 6.52)	42.8 (42.2 - 43.4)	57.2 (56.5 - 57.9)

2014	646	6.27 (6.12 - 6.43)	41.2 (40.6 - 41.9)	58.8 (58.1 - 59.5)
2015	646	6.22 (6.07 - 6.37)	41.1 (40.8 - 41.4)	58.9 (58.6 - 59.2)
2016	561	6.23 (6.08 - 6.38)	40.9 (40.6 - 41.2)	59.1 (58.8 - 59.5)
2017	505	6.13 (5.95 - 6.30)	39.7 (39.4 - 40.0)	60.3 (60.0 - 60.7)
2018	450	6.09 (5.92 - 6.25)	37.7 (37.3 - 38.0)	62.3 (62.0 - 62.7)
P for trend		<0.001*	<0.001*	<0.001*
P for stratified variable AWS*round		0.743	0.741	0.741
**Females**				
Total				
2011	2916	6.06 (5.97 - 6.15)	46.0 (45.6 - 46.5)	54.0 (53.5 - 54.4)
2012	2087	6.16 (6.07 - 6.25)	40.7 (40.2 - 41.1)	59.3 (58.8 - 59.9)
2013	1670	6.09 (5.99 - 6.19)	38.0 (37.5 - 38.5)	62.0 (61.5 - 62.4)
2014	1368	6.12 (6.02 - 6.23)	37.9 (37.4 - 38.4)	62.1 (61.6 - 62.6)
2015	1374	5.90 (5.79 - 6.01)	36.5 (36.4 - 36.7)	63.5 (63.3 - 63.7)
2016	1222	5.92 (5.78 - 6.07)	35.0 (34.7 - 35.2)	65.0 (64.8 - 65.3)
2017	1087	5.88 (5.76 - 6.01)	35.2 (35.0 - 35.4)	64.8 (64.5 - 65.1)
2018	957	5.89 (5.76 - 6.02)	34.7 (34.4 - 34.9)	65.3 (65.1 - 65.6)
P for trend		<0.001*	<0.001*	<0.001*
Normal				
2011	248	6.63 (6.39 - 6.87)	62.7 (61.2 - 64.3)	37.3 (36.1 - 38.5)
2012	174	6.61 (6.37 - 6.84)	50.4 (49.1 - 51.7)	49.6 (48.0 - 51.1)
2013	138	6.74 (6.48 - 6.99)	50.8 (49.6 - 52.1)	49.2 (48.0 - 50.4)
2014	113	6.96 (6.66 - 7.25)	58.4 (57.0 - 59.9)	41.6 (40.2 - 43.0)
2015	111	6.86 (6.61 - 7.11)	57.7 (57.2 - 58.2)	42.3 (41.7 - 42.9)
2016	96	6.68 (6.40 - 6.97)	53.4 (52.9 - 53.9)	46.6 (46.0 - 47.2)
2017	86	6.72 (6.40 - 7.03)	54.7 (54.0 - 55.3)	45.3 (44.5 - 46.1)
2018	81	6.69 (6.31 - 7.07)	61.1 (60.3 - 61.8)	38.9 (38.2 - 39.6)
P for trend		<0.001*	<0.001*	<0.001*
Overweight				
2011	472	6.59 (6.40 - 6.78)	59.6 (58.6 - 60.6)	40.4 (39.4 - 41.4)
2012	338	6.74 (6.59 - 6.89)	52.1 (51.3 - 53.0)	47.9 (46.9 - 48.9)
2013	298	6.67 (6.48 - 6.86)	54.5 (53.5 - 55.4)	45.5 (44.4 - 46.6)
2014	241	6.72 (6.52 - 6.91)	53.8 (52.8 - 54.8)	46.2 (45.1 - 47.4)
2015	244	6.47 (6.30 - 6.65)	52.2 (51.8 - 52.7)	47.8 (47.3 - 48.3)
2016	219	6.59 (6.39 - 6.80)	53.4 (52.9 - 53.9)	46.6 (46.1 - 47.1)
2017	199	6.40 (6.11 - 6.68)	48.1 (47.6 - 48.5)	51.9 (51.3 - 52.5)
2018	169	6.54 (6.31 - 6.76)	45.6 (45.1 - 46.1)	54.4 (53.8 - 55.0)
P for trend		<0.001*	<0.001*	<0.001*

Obese				
2011	2196	5.87 (5.75 - 5.98)	40.8 (40.2 - 41.3)	59.2 (58.6 - 59.8)
2012	1575	5.98 (5.87 - 6.08)	36.8 (36.3 - 37.3)	63.2 (62.6 - 63.7)
2013	1234	5.86 (5.75 - 5.98)	32.6 (32.1 - 33.1)	67.4 (66.9 - 67.9)
2014	1014	5.87 (5.76 - 5.98)	31.8 (31.3 - 32.2)	68.2 (67.8 - 68.6)
2015	1019	5.64 (5.51 - 5.78)	30.4 (30.2 - 30.6)	69.6 (69.3 - 69.8)
2016	907	5.67 (5.52 - 5.81)	28.4 (28.2 - 28.6)	71.6 (71.4 - 71.9)
2017	802	5.65 (5.51 - 5.79)	29.8 (29.6 - 30.0)	70.2 (69.9 - 70.5)
2018	707	5.63 (5.49 - 5.77)	28.9 (28.6 - 29.1)	71.1 (70.8 - 71.4)
P for trend		<0.001*	<0.001*	<0.001*
P for stratified variable AWS*round		0.099	0.497	0.497

Note: [@]p for trend were estimated from univariate mixed model, and [&]p for trend were estimated from generalized estimating equation model accounting for the correlation with repeated measures, utilizing weighted data with the follow-up round analytic weight which adjusts for loss to follow-up and round was treated as continuous variable (1-8). AWS = abdominal weight status; NHATS = National Health and Aging Trends Study; HAS = healthy aging score. [#]Good HAS defined as above the median whereas poor HAS defined as below the median; *p<0.05.
